# Formation of spherical Sn particles by reducing SnO_2_ film in floating wire-assisted H_2_/Ar plasma at atmospheric pressure

**DOI:** 10.1038/s41598-020-74663-z

**Published:** 2020-10-20

**Authors:** Thi-Thuy-Nga Nguyen, Minoru Sasaki, Takayoshi Tsutsumi, Kenji Ishikawa, Masaru Hori

**Affiliations:** 1 Nagoya University, Nagoya, 464-8601 Japan; 2 Toyota Technological Institute, Nagoya, 468-8511 Japan

**Keywords:** Electrical and electronic engineering, Chemical physics, Plasma physics, Design, synthesis and processing

## Abstract

A green method to synthesize spherical Sn particles by reducing SnO_2_ film in atmospheric-pressure H_2_/Ar plasma at low temperatures for various applications is presented. The floating wire-assisted remotely-generated plasma with a mixture of 0.05% H_2_/Ar gas formed spherical metallic Sn particles by reducing a SnO_2_ layer on glass substrate. During the reduction process, H radical density was measured by using vacuum ultraviolet absorption spectroscopy, and plasma properties including electron density and gas temperature were diagnosed by optical emission spectroscopy. The inductively coupled generated plasma with a high electron density of 10^14^ cm^−3^, a hydrogen atom density of 10^14^ cm^−3^, and a gas temperature of 940 K was obtained at a remote region distance of 150 mm where the SnO_2_/glass substrate was placed for plasma treatment. The process has been modeled on the spherical Sn formation based on the reduction of SnO_2_ films using H radicals. Depending on the treatment condition, the total reduction area, where spherical Sn particles formed, was enlarged and could reach 300 mm^2^ after 2 min. The substrate temperature affected the expansion rate of the total reduction area and the growth of the Sn spheres.

## Introduction

Tin (Sn) metal has been extracted from ores for a long time^1,2^, and is a highly demanded material for different industrial applications including Pb-free solder^3,4,5^, batteries^6–8^, and transparent electrodes^9,10^. Removal of Sn contamination on extreme ultraviolet (EUV) lithography optics can be applied by using H radicals^11–14^. Moreover, Sn nano/micro-particles embedded in SiO_2_ matrix or Sn-implanted SiO_2_ structure are potentially applied in development of novel devices^15,16^.

The reduction of SnO_2_ can form Sn metal by various gases, such as CH_4_ gas^17^ and H_2_ gas^18^, with high-temperature treatment required during the reduction process. Among these gases, the reduction of SnO_2_ by H_2_ gas can extract Sn metal without CO_2_ emission that causes global warming^18^. RF plasma-decomposed hydrogen from pure H_2_ gas was used to reduce SnO_2_^19^. The hot wire method using a wire temperature of 1850 °C and radio frequency (RF) powered plasma were used to reduce SnO_2_ by generating H radical from pure H_2_ gas^19^. Substrate temperature (T_sub_) of 430 °C and minimum treatment time of 10 min were required to form granule-like particles of metallic Sn^19^. H radicals play the most important role during the reduction process; nevertheless, the data for the presence of H radicals or the measurement of H radical density as well as the mechanism of the reduction process to form Sn have not been clarified yet.

In comparison with thermal plasma that has an extremely high temperature (~ 10,000 K) and has been applied in synthesizing high-purity metals, especially for refractory metals and high-temperature resistant metals^20–23^, there are a few studies using low-temperature atmospheric-pressure plasma to synthesize metals. For example, the low-temperature plasma, that only electrons have high temperature, could synthesize granular shapes of metallic Cu nanoparticles from a Cu wire at a gas temperature of 1500 K^24,25^. In addition, the non-thermal plasma can also reduce copper oxides on copper^26^. In our previous study, we developed a remotely floating-wire-assisted plasma source that can generate plasma with a low gas temperature (< 1000 K) and a high-plasma density (electron density of 10^14^ cm^−3^) under atmospheric pressure^27^. The long floating wire (> 130 mm) could transport a high-density atmospheric-pressure inductively couple plasma (AP-ICP) to a remote region at the downstream due to a high electric field generated near the end of the floating wire. This plasma source has a potential to reduce SnO_2_ films in a large area by using H_2_-based plasma technology.

This study aims to develop a green method to synthesize spherical Sn particles by reducing SnO_2_ film in atmospheric-pressure H_2_/Ar plasma at low temperatures. The synthesis of spherical Sn particles via a reduction process from SnO_2_ film on glass substrate by low-temperature AP-ICP (< 1000 K) was revealed in the H_2_/Ar plasma with a very low H_2_ gas concentration of 0.05% instead of pure H_2_ gas at low T_sub_ (~ 100 °C). Plasma properties were diagnosed by optical emission spectroscopy (OES). H radical density was measured by using vacuum ultraviolet absorption spectroscopy (VUVAS)^28–30^. The treatment time and T_sub_ affect the expansion rate of the total reduction area and the growth of the spherical Sn particles. A model for the formation of spherical Sn particles from SnO_2_ film on glass substrate in the atmospheric-pressure H_2_/Ar plasma is presented in this paper.

## Results and discussion

### Reduction of a SnO_2_ film with the formation of Sn particles at low T_sub_ (≤ 150 °C)

**Figure 1:**
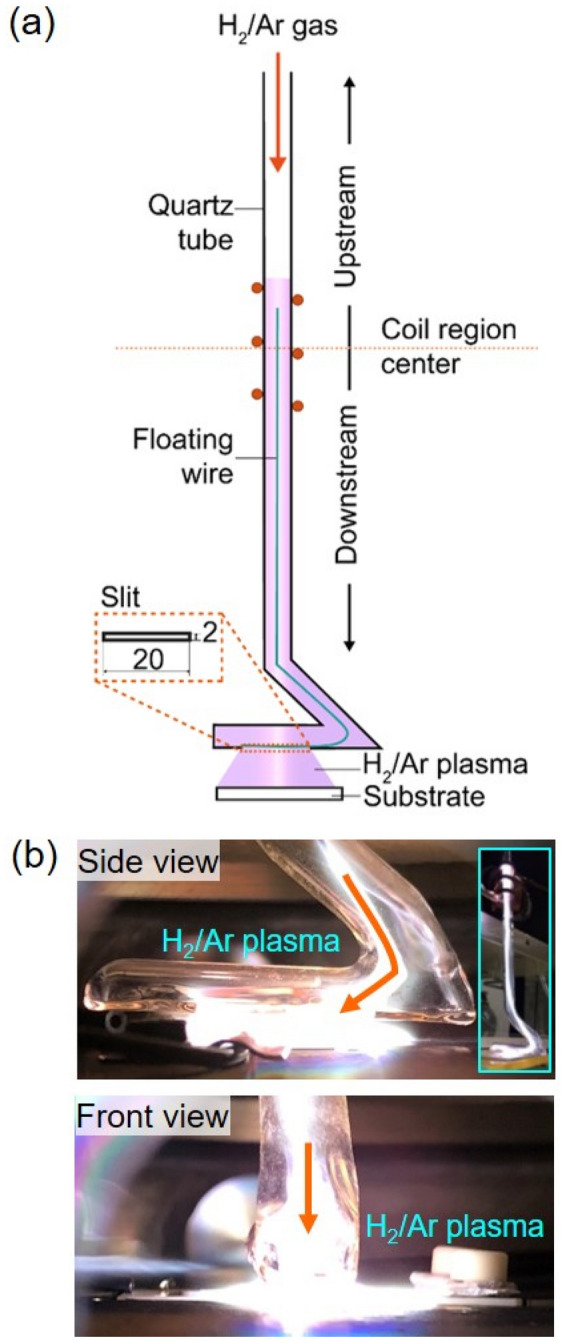
(**a**) Schematic of the floating wire-assisted AP-ICP discharge using H_2_/Ar gas. (**b**) H_2_/Ar plasma generated inside the chamber observed from side view and front view.

An inductively coupled long floating wire inside the quartz tube could transport high-density plasma at a remote region of 150 mm, as shown in Fig. 1a. By generating the electric field at the end of floating wire, the bright plasma blew out from the slit of L-shaped discharge tube at the remote downstream region (Fig. 1b), as discussed previously^27^. The pristine SnO_2_ film/glass substrate was placed at the remote region for plasma treatment.

**Figure 2:**
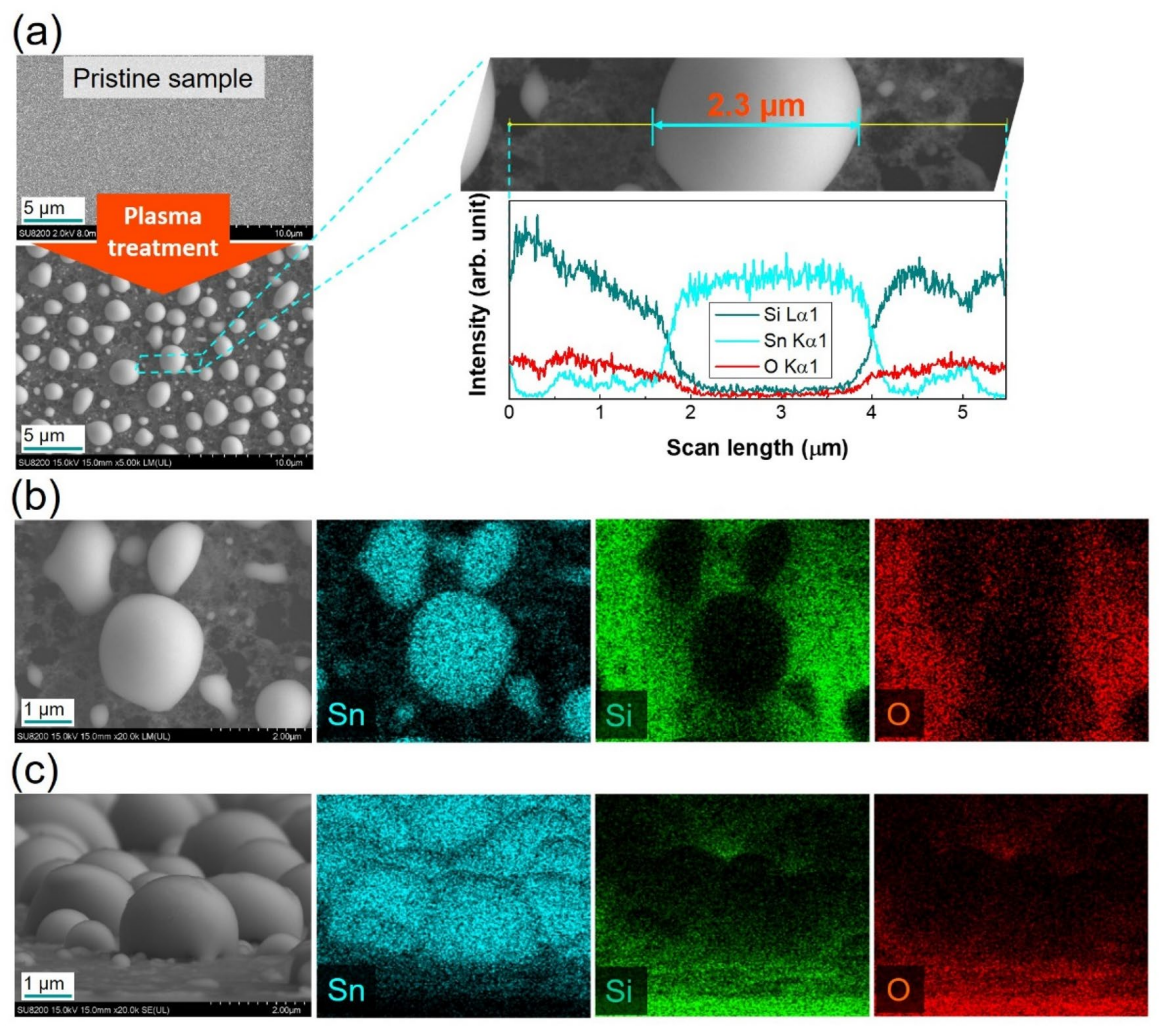
(**a**) Surface morphologies, and line profiling analysis of pristine sample and treated sample without using heater. (**b**) Top-view elemental mapping images of partly reduced SnO_2_ area on glass substrate. (**c**) Tilted-view elemental mapping images of highly reduced SnO_2_ area on glass substrate.

In Fig. 2a, a glassy surface was observed before plasma treatment, and the metallic Sn islands were observed in the top-view SEM image and line profiling analysis after plasma treatment. Details of top-view elemental mapping images are shown in Fig. 2b with Sn islands formed on SnO_2_ film. In Fig. 2c, tilted-view elemental mapping images of treated sample at a higher reduction rate were taken. Nearly spherical Sn particles were formed on glass surface (see Sn element image) while the SnO_2_ (see O element image) was remained as a very thin layer.

**Figure 3:**
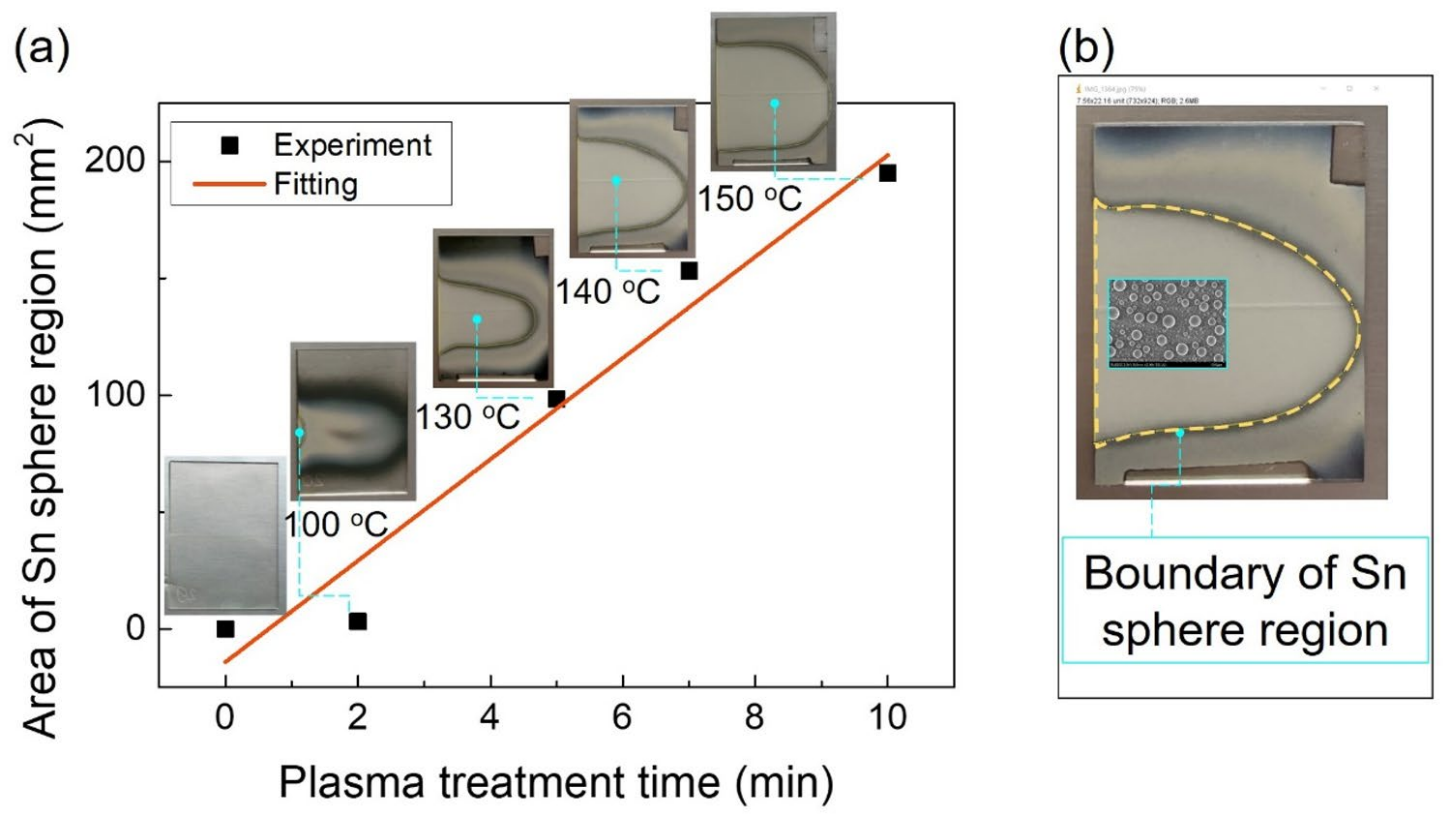
(**a**) Area of total reduction region (Sn sphere region) as a function of plasma treatment time. Inset: photographs of SnO_2_/glass substrate treated from 0 min (pristine sample) to 10 min. (**b**) Photograph image of the boundary of Sn sphere region (yellow dashed line) after 7 min treatment.

The total reduction areas, where the spherical Sn particles formed, were linearly expanded when the plasma treatment time increased (Fig. 3). In the photograph images (Fig. 3a), the pristine sample is highly transparent, and the treated sample first turned to black then opaque white. When the opaque film was totally reduced with the formation of Sn spheres, the surface became more transparent. Figure 3b shows a photograph of the treated sample. A dashed yellow line depicts the boundary of the total reduction region, where the Sn spherical particles formed. The area of the totally reduced region reaches 200 mm^2^ after 10 min treatment. The T_sub_ of samples during the plasma treatment was recorded by a thermocouple. This T_sub_ obtained from only H_2_/Ar plasma (without using any additional heater) and gradually increased with the treatment time from 100 °C (2 min) to 150 °C (10 min). The T_sub_ also affects to the reduction area. This will be discussed later (Fig. 6).

**Figure 4:**
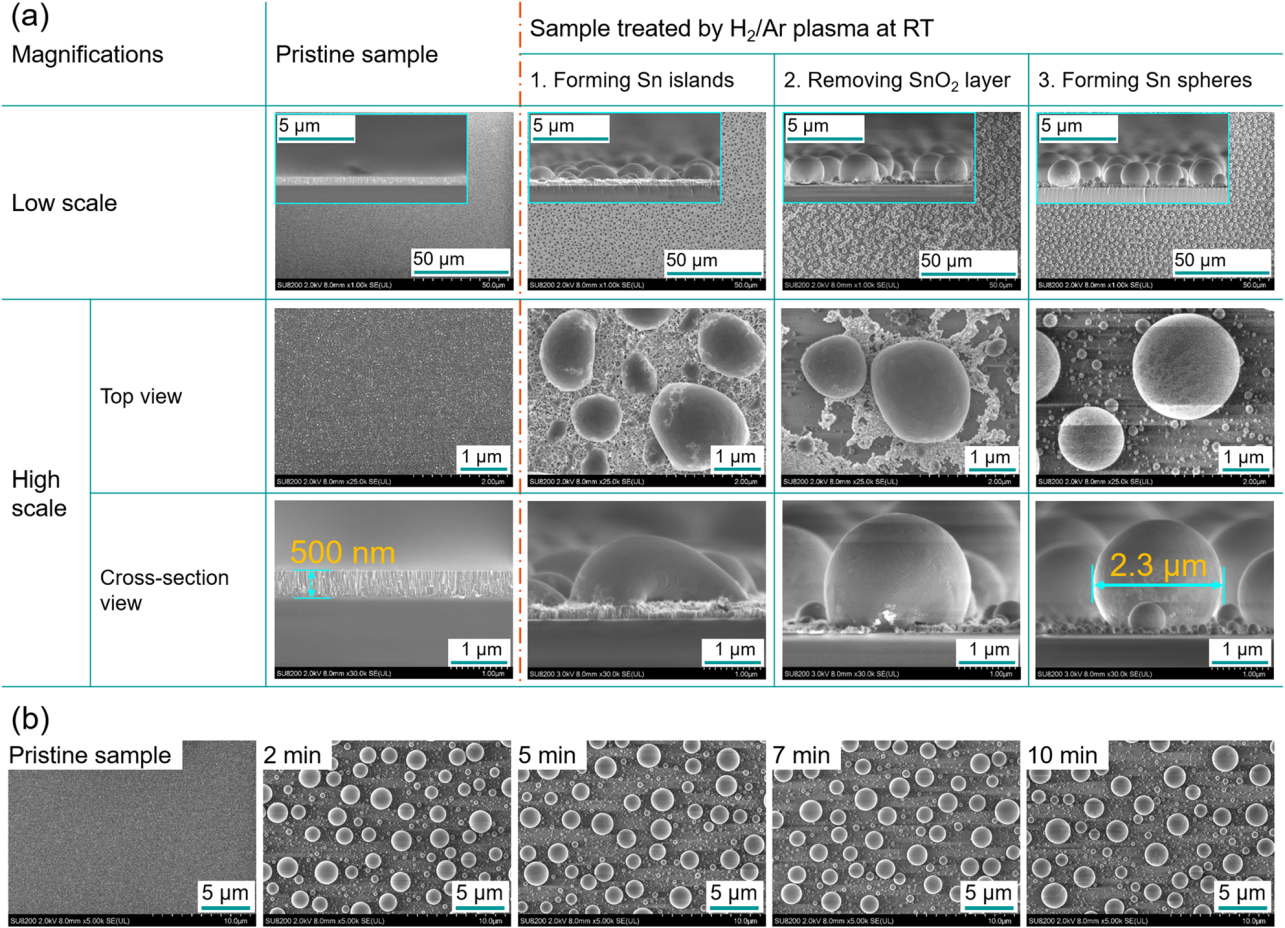
(**a**) Reduction process of SnO_2_/glass substrate by H_2_/Ar plasma treatment at low temperature. (**b**) Surface morphology of pristine sample (left) and samples treated from 2 to 10 min (right) at low T_sub_.

Figure 4a presents the reduction process of SnO_2_/glass substrate by H_2_/Ar plasma treatment without using any additional heater. The pristine sample has a 500-nm-thick SnO_2_ film. A glassy surface and a dense column structure of the SnO_2_ film can be seen in the top-view and cross-sectional images, respectively. After treated with H_2_/Ar plasma, the reduction process was classified into three steps: (1) forming Sn islands, (2) removing SnO_2_ layer, and (3) forming Sn spheres. At the step 1, the island-like Sn with diameters up to several µm was covered on the remained SnO_2_ film surface. The height of the Sn islands grew more than 1 µm that is much higher than that of pristine sample (500 nm). At the step 2, the SnO_2_ film became thinner and was partly disappeared, and the shape of the Sn particles was more rounded. At the step 3, the SnO_2_ film was disappeared for forming of nano- and micro-meter sized Sn spherical particles. The shape of the Sn particles changed to a true sphere. This can be interpreted by changing interfacial energy. The underlying surface of the Sn metal was changed from SnO_2_ to glass with different surface tensions. In other words, the contact angle of Sn particles with substrate also changed from step 1 (< 80°) to step 2 (80–130°) and to step 3 (> 130°). The Sn contact angles at step 1, step 2, and step 3 are shown in cross-sectional SEM images of plasma-treated SnO_2_/glass substrate (Figure S1).

The totally reduced region (white area, marked 'R1′ with the points 1 and 2 in Figure S2) consists of nano and micro Sn spherical particles formed on glass substrate after 5 min plasma treatment without using heater. The area of spherical Sn particles (around 100 mm^2^) is one third of the whole SnO_2_ film surface (300 mm^2^). The highly reduced region (black area, marked ‘R2’ with the points 3 and 4 in Figure S2) contains nearly spherical or oval-shaped Sn particles. Some SnO_2_ areas left under these particles that need more H radicals for total reduction. The partly reduced region (grey area, marked ‘R3’ with the points 5–7 in Figure S2) includes island-like Sn surface and SnO_2_ films simultaneously. The size of the Sn islands on thick SnO_2_ film was determined by the reduction rates.

Dynamical changes in the surface morphology of pristine sample and samples treated from 2 to 10 min for total reduction region (Sn sphere region) at low T_sub_ are exhibited in Fig. 4b. The diameter of Sn spheres was slightly increased with the longer treatment time.

### Electron density of floating wire-assisted H_2_/Ar plasma at remote region

**Figure 5:**
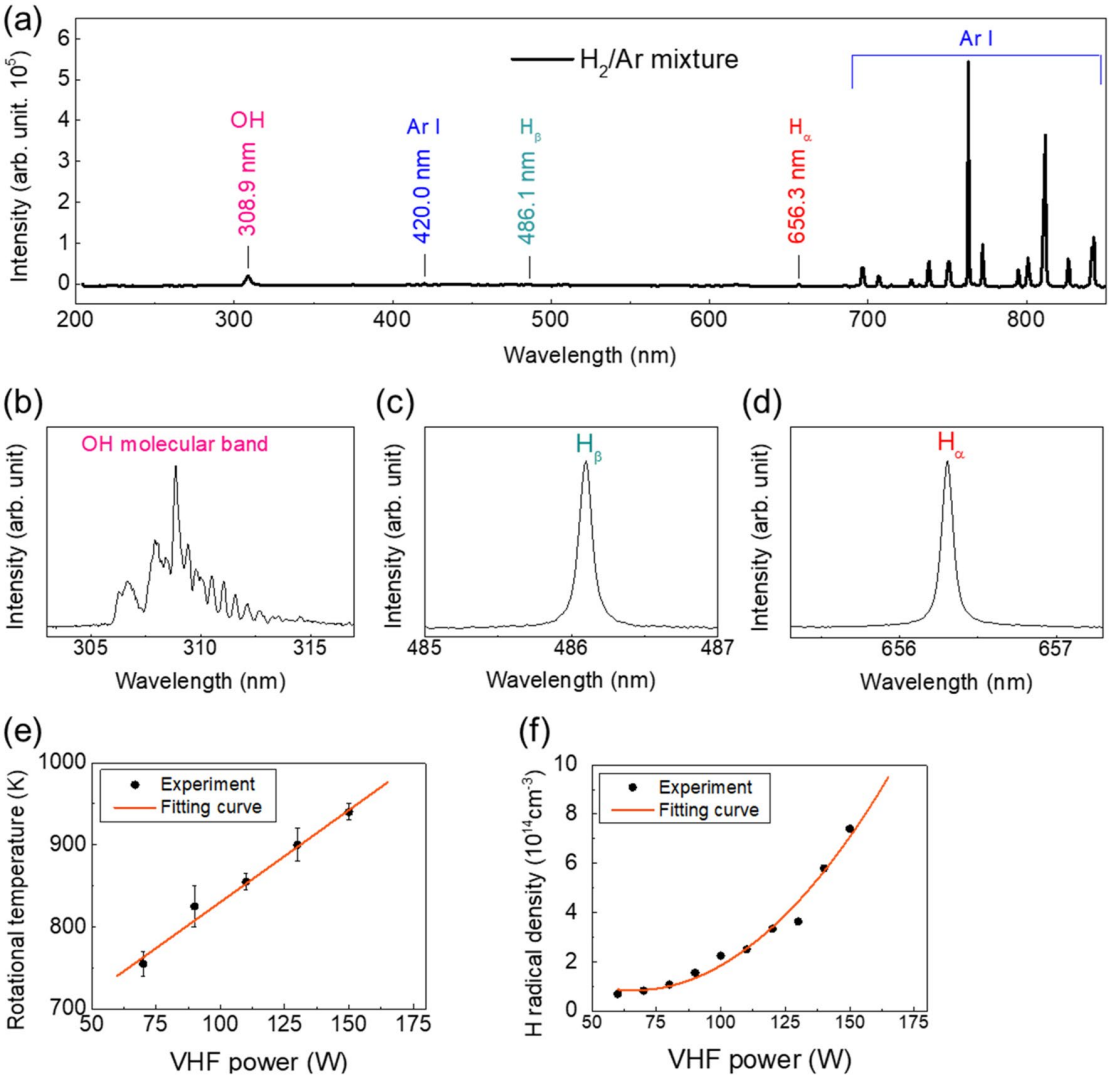
Emission spectra of (**a**) H_2_/Ar plasma generated at 150 W. Emission spectra of (**b**) OH molecular band (**c**) H_β_ (**d**) H_α_. (**e**) Gas temperature of H_2_/Ar plasma as a function of VHF power. (**f**) H radical density as a function of VHF power.

In order to clarify the mechanism of the reduction process as well as the formation of spherical Sn particles at low T_sub_, the properties of floating wire-assisted H_2_/Ar plasma source were presented in Fig. 5. Figure 5a shows optical emission spectra of the floating wire-assisted remote H_2_/Ar plasma generated at 150 W. The OH molecular band (band head at 308.9 nm), H_α_ emission (656.3 nm), and H_β_ emission (486.1 nm) can be detected from the spectra for H_2_/Ar gas. Enlarged spectra of OH molecular band (Fig. 5b), H_β_ emission (Fig. 5c), and H_α_ (Fig. 5d) emission are presented. The electron density was determined by using Stark broadening width of H_β_^27,31,32^. The optical emission spectrum of H_β_ in H_2_/Ar plasma was fitted by the simulation spectrum with the Voigt function, and Stark width equals to Lorentzian part of Voigt profile. The measured electron density of H_2_/Ar at 150 W is around 4 × 10^14^ cm^−3^ (Figure S3).

### Gas temperature

OH molecular band shown in Fig. 5b is from the product of the reduction process between SnO_2_ with H radicals and from the moisture remained inside the chamber. In order to evaluate gas temperature of H_2_/Ar plasma, it was assumed as rotational temperature (T_r_) of OH molecular band (A^2^∑^+^, ν = 0)^33,34^. By using the OES data of OH molecular band produced from H_2_/Ar plasma at 150 W (dot line) and the fitting simulated spectrum (solid line) done by LIFBASE program, the best fit of the simulated spectrum at 940 K was obtained (Figure S4). The plasma properties including gas temperature and H radical density were determined at various VHF powers. The gas temperature gradually increases from around 750 to 1000 K with increasing power from 70 to 175 W for when using H_2_/Ar gas (Fig. 5e).

### Hydrogen radical density

The exited hydrogen atoms or H radicals can be produced by the collision between H_2_ molecules and electrons and Ar metastable atoms. The H radicals are easily produced by Ar metastable atoms because the dissociation energy of H_2_ molecules (~ 4.5 eV) is lower than the minimum excitation energy (~ 11.6 eV) and ionization energy (~ 15.75 eV) of Ar atoms^24,25^.

The absolute H radical density was calculated in the order of 10^14^ cm^−3^ (Fig. 5f). When VHF power increases from 60 to 150 W, an increase can be obtained in H radical density. When VHF power is more than 80 W, the H radical density reaches 10^14^ cm^−3^. H radical density is 7 × 10^14^ cm^−3^ at 150 W. A very high rate of H radical (1–10%) could be produced from this plasma source with a very low concentration of H_2_ (0.05%) in the H_2_/Ar mixture.

The absolute H radical density was calculated in the order of 10^14^ cm^−3^, resulting a capability for a high reduction rate by applying this plasma source for various plasma treatment applications.

### Influence of T_sub_ on the reduction of SnO_2_ film

Without using any additional heater, the T_sub_ gradually increased with the treatment time from 100 °C (2 min) to 150 °C (10 min) (Fig. 3). At the totally reduced region, the size of spherical particles presents a slight increase from 2 to 10 min plasma treatment.

**Figure 6:**
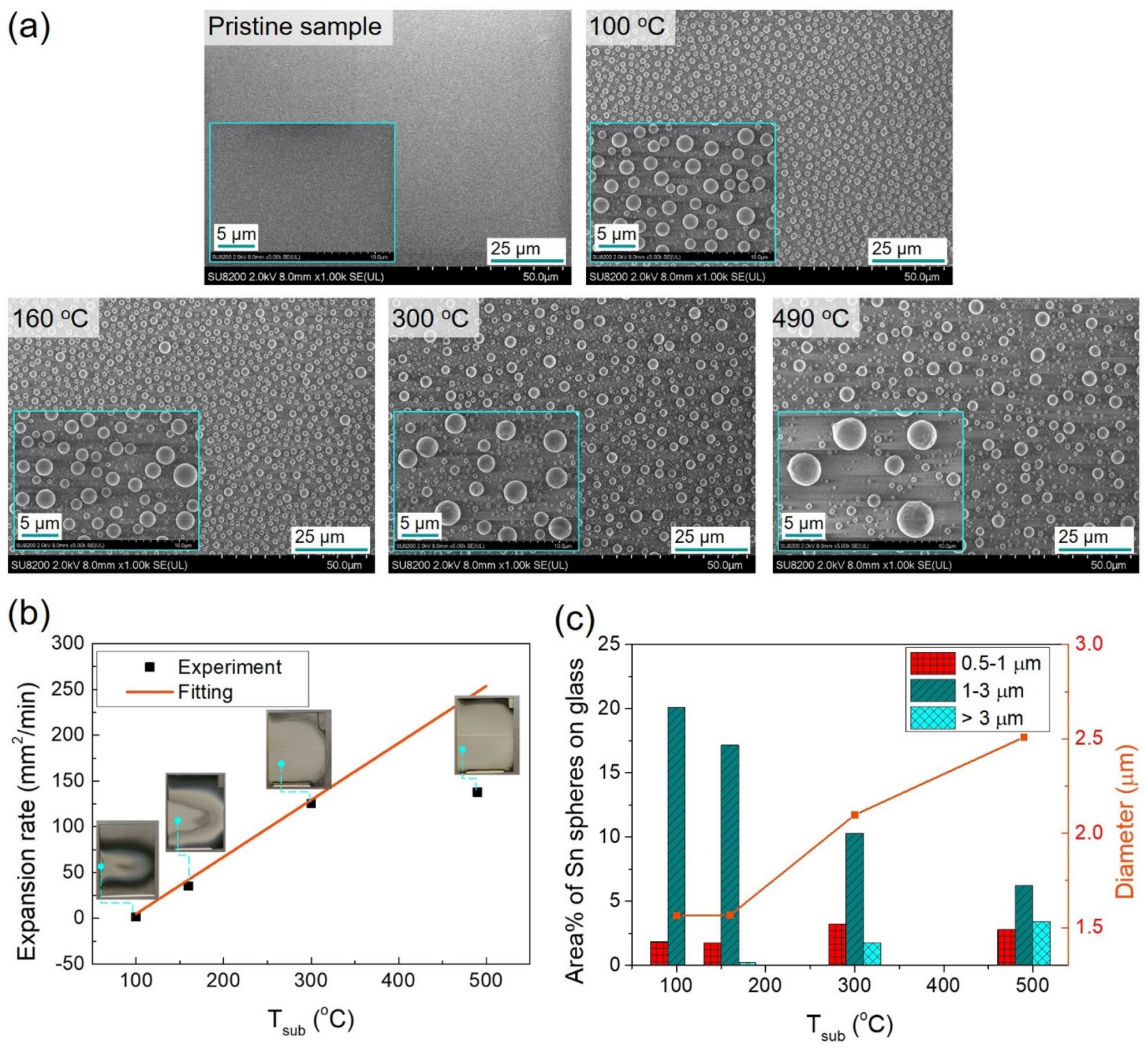
(**a**) Top-view SEM images of pristine sample and 2-min-treated samples at T_sub_ of 100 °C, 160 °C, 300 °C, and 490 °C. (**b**) Expansion rate of total reduction area (Sn spheres area) as a function of T_sub_. Inset: photographs of SnO_2_/glass substrates treated at various T_sub_ after 2 min. (**c**) Covering area percentage of Sn spheres on glass and Sn spherical diameter at various T_sub_.

In order to compare the reduction of SnO_2_ film by AP-ICP at low temperature with that at high temperature, the T_sub_ was increased by using an additional heater. Figure 6 shows the influence of T_sub_ on surface morphology of treated samples, the expansion rate of total reduction area, the covering area ratio of Sn spheres on glass, and the Sn diameter change after 2 min plasma treatment. Top-view SEM images of pristine sample and samples treated from 100 °C (the sample in Fig. 4b without using any heater, the T_sub_ is from plasma only) to 490 °C are shown in Fig. 6a. Larger Sn spherical particles were formed with an increase of T_sub_, whereas the number of Sn spherical particles was reduced. When the temperature increases from 100 to 160 °C, the Sn sphere diameter was insignificantly increased. This agrees with the results from Fig. 4b in which the T_sub_ increases from 100 °C (by 2 min treatment) to 150 °C (by 10 min treatment). The particle size increases more obviously when the temperature raises from 160 to 300 °C, and the number of Sn particles also reduces clearly. When the T_sub_ was 490 °C, the particle diameter can reach up to 2.5 µm in an average size (Fig. 6c), and particles with the diameter more than 5 µm can be observed from the SEM image in Fig. 6a.

Figure 6b shows the calculated expansion rate of the total reduction area (Sn sphere area). The T_sub_ significantly improved the expansion rates of the reduced areas after 2 min plasma treatment. The reduction area expands almost 3.5–4 times when T_sub_ increases from 160 to 300 °C and to 490 °C. The reduction rate was nearly 150 mm^2^/min at 490 °C. The diameter of Sn spheres was a mean value for micro-particles (Fig. 6c). When the Sn sphere diameter increases from 1.6 µm (100 °C) to 2.5 µm (490 °C), the area of Sn spheres covering glass substrate reduces from 22 to 12%.

In comparison with samples treated at a high temperature using the same H_2_/Ar mixture without plasma ignition, the reduction rate is very low (Figure S5). The sample was exposed under the same H_2_/Ar mixture with the same flow rate (6 standard liter per minute (slm)) and T_sub_ was 460 °C. After 2 min (Figure S5b) and 20 min (Figure S5c) exposing under H_2_/Ar mixture, no significant change can be observed on the sample surface. Very few nano Sn particles (around 60–70 nm) formed on the whole SnO_2_ surface. In contrast, the samples treated by H_2_/Ar plasma at both low temperature (Figure S5d) and high temperature (Figure S5e) show the formation of nano Sn spheres and micro Sn spheres (up to 5 µm in diameter).

### Model of forming spherical Sn particles by H_2_/Ar plasma

**Figure 7:**
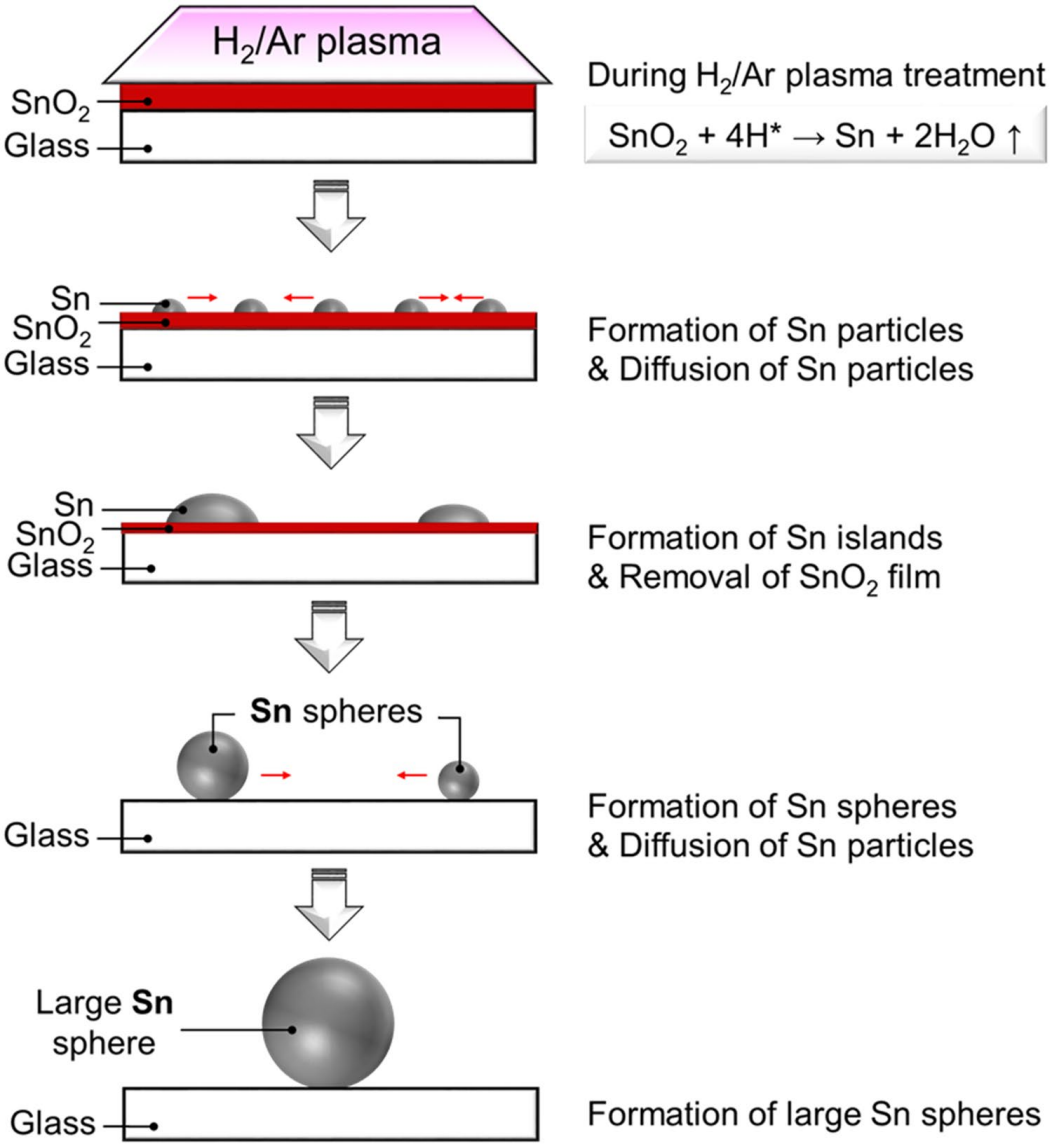
Model of forming Sn spherical particles by reducing SnO_2_ film during the H_2_/Ar plasma treatment.

The model of forming spherical Sn particles during the atmospheric-pressure plasma treatment is proposed in Fig. 7. High-density floating wire-assisted H_2_/Ar plasma (electron density ~ 10^14^ cm^−3^) produced high H radical density (~ 10^14^ cm^−3^) and a gas temperature of 940 K. The H radicals produced from H_2_/Ar plasma react with SnO_2_ as follows:

1 $${\text{SnO}}_{2} + {\text{4H}}^{*} \to {\text{Sn}} + {\text{2H}}_{2} {\text{O}}$$

The H radicals (H^+^ may also produce in the plasma) captured the oxygen in the SnO_2_ film. Sn particles gradually formed, whereas SnO_2_ film reduces its thickness. Since the gas temperature of H_2_/Ar plasma (940 K) is much higher than the melting temperature of a Sn metal (231.9 °C)^35^, the reduction process can occur and form the Sn clusters even without using any heater. When T_sub_ of the H_2_/Ar plasma increased, melted Sn particles agglomerated to form a larger Sn surface.

By removing SnO_2_ film on glass surface, the surface energy that is underlying Sn particles was also reduced and stabilized, which forms spherical Sn particles. Under the effect of surface tension, the small melted Sn clusters were condensed to form spherical particles. Spherical Sn formed with reducing its contact area to glass substrate, resulting less covered area of Sn on glass while no SnO_2_ film left. Therefore, formation of Sn spheres occurred under plasma treatment of the SnO_2_ film on glass substrate. A similar effect for the spheroidization of the irregular molybdenum powder to form high-purity micro-molybdenum powders by thermal RF plasma was discussed in Liu et al^23^. This process required a thermal plasma torch having an extremely high temperature (~ 10,000 K) with a rapidly cooled tail (10^5^–10^6^ K/s) at 100 kW, 4 MHz. The high temperature region can provide enough energy for the melting/evaporation of the raw materials and the rapidly cooled tail could help rapid solidification. Both nano and micro Sn particles were generated after plasma treatment. This could be explained by the fact that melting-spheroidization and evaporation–condensation coexist during the formation of spherical particles. Melting–spheroidization results in micron spheres while evaporation–condensation leads to spherical nanoparticles^20^. Formation of Sn spherical particles can reduce its surface area, reducing the Sn surface area exposing to the air, and hence, it minimizes the re-oxidation problem.

The influence of T_sub_ on the growth of Sn spheres can be explained by two reasons. The first is that gas temperature of plasma itself was 940 K that is higher than the melting temperature of Sn particles. Combining with a rich H radical source, Sn clusters can be formed from agglomeration of small melted Sn particles at low T_sub_ (Fig. 3). The latter is that the expansion rate of total reduction area and the Sn diameter increase significantly as the T_sub_ increases from 160 to 300 °C. The Sn particle size became larger at high T_sub_ (> 300 °C). Substantially, the size of the Sn particles distributed in nanometer and micrometer range. In comparison with the micro Sn particles, the nano Sn particles have a lower melting temperature and higher diffusion velocity^36^. Higher T_sub_ leads faster surface diffusion velocity of small Sn particles, resulting in the longer diffusion length, that forms larger Sn particles.

On the large Sn sphere surface, a recombination of H radical generates heat as follows:

2 $${\text{H}}^{*} + {\text{ H}}^{*} \to {\text{H}}_{{2}}$$

The reaction (2) is an exothermic reaction (enthalpy ΔH = − 436 kJ/mol at 298 K and 1 bar)^37,38^. An increase in a net temperature combined between the recombination heat and T_sub_ enhances Sn etching reaction as follows:

3 $${\text{Sn }} + {\text{ 4H}}^{*} \to {\text{ SnH}}_{{4}} \uparrow$$

**Figure 8:**
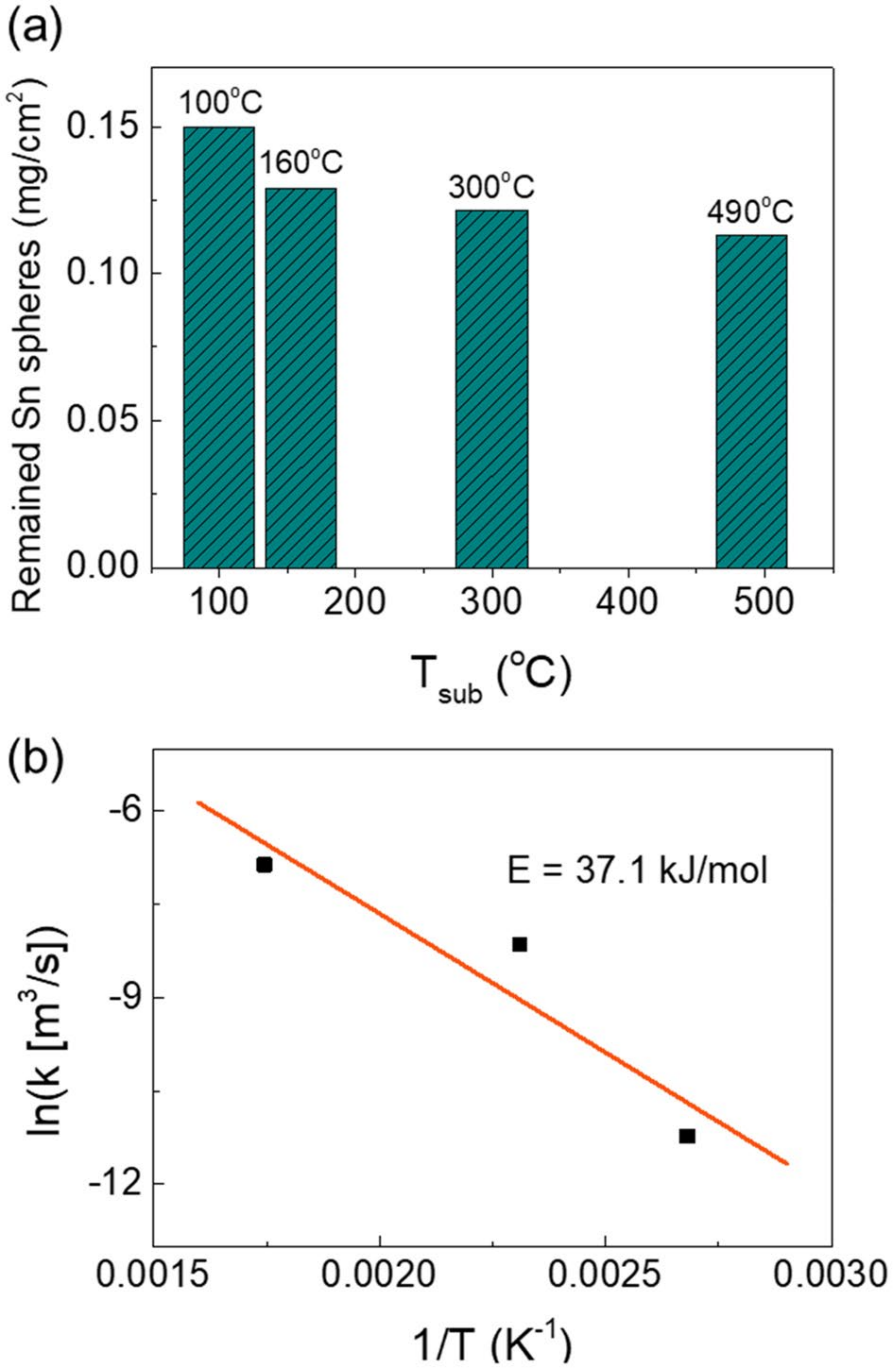
(**a**) Dependence of mass of remained Sn spheres calculated at the same total reduction area (1 cm^2^) on T_sub_. (**b**) An Arrhenius plot of the total reduction rate constant at various T_sub_.

To clarify the reaction (3), the influence of T_sub_ on the mass of remained Sn spheres on glass after 2-min H_2_/Ar plasma treatment calculated at the same total reduction area (1 cm^2^) is shown in Fig. 8a. The mass of remained Sn spheres decreases with an increase of T_sub_ from 0.15 mg/cm^2^ (100 °C) to 0.11 mg/cm^2^ (490 °C). Partly of Sn was etched by the reaction (4). In addition, the roughened surface of Sn spheres was observed at high T_sub_ as a result of the etching reaction (3) (Fig. 6a).

Based on the results from the expansion rate of total reduction area (Sn spheres area) in Fig. 6b, the values of the total reduction rate constant, k, was obtained by the following equation:

4 $${\text{k }} = {\text{ expansion rate of total reduction area }} \times {\text{ Sn film thickness }}\left[ {{\text{mm}}^{{3}} /{\text{s}}} \right]$$

An Arrhenius plot of the total reduction rate constant k at various T_sub_ is presented in Fig. 8b. The slope of the fitting line corresponds to an activation energy of 37.1 kJ/mol that is the minimum energy for occurring reduction reaction and forming Sn spheres. The fitting line can be expressed as follows:

5 $${\text{k }} = { 3}.{58} e^{{ - 4461.75/{\text{T}}}} \left( {{\text{mm}}^{{3}} /{\text{s}}} \right)$$

In order to remove Sn spheres from glass surface, it is also important to remove the thin residual SnO_2_ layer that is underlying Sn spheres by only H_2_/Ar plasma treatment. The SnO_2_ which is beneath smaller Sn spheres has a smaller contact area in comparison with that under large Sn spheres, which facilitates the penetration of H radical and promotes to react with SnO_2_. The small Sn particles could be removed by gas flow (6 slm) and chamber pumping during the plasma treatment. Once SnO_2_ was removed from the glass surface, the bonding strength between Sn particles and glass surface loosened, resulting in a removal of Sn particles from glass surface. Therefore, Sn can be etched by floating wire-assisted H_2_/Ar plasma, this can be applied to remove the Sn contamination on EUV optics of EUV lithography systems^11–14^.

The floating wire-assisted H_2_/Ar plasma source has advantages to form spherical Sn particles by reducing SnO_2_ at a low substrate temperature (T_sub_), as follows:

1. In comparison with vacuum plasma sources, this floating wire-assisted atmospheric plasma source can miniaturize of equipment size and reduce fabrication cost and energy consumption.
2. Rich H radicals can be provided by this plasma source although very low H_2_ gas concentration (0.05% H_2_/Ar) is used instead of using pure H_2_ gas or CF_4_ gas to reduce SnO_2_ to form Sn particles. A green method to synthesize spherical Sn particles was proposed in this paper, in which no toxic chemical was used, and no CO_2_ emission that causes global warming was released.
3. Spherical Sn particles can be formed after H_2_/Ar plasma treatment at a low T_sub_ (~ 100 °C) without using any additional heater.

## Conclusions

With the floating wire-assisted remote plasma generation, 0.05% H_2_-added-Ar plasma was used to generate high H radical density up to 10^14^ cm^−3^ and gas temperature of 940 K to form the island-like Sn structure (partial reduction) and Sn spheres (total reduction) on glass substrate at low T_sub_ (without using any heater). The plasma source properties, such as gas temperature and H radical density, were measured by using OES and VUVAS techniques, respectively. Larger Sn spheres and higher reduction rate can be obtained at higher T_sub_ (more than 300 °C). The study opens a wide range of applications for the low-temperature atmospheric-pressure plasma source such as the extraction of low-melting point metals, the synthesis of high-purity metal spheres, and the removal of contamination containing metals or metal oxides.

## Materials and methods

### Sample preparation

SnO_2_/glass samples with SnO_2_ film deposited were provided by AGC Inc. The thicknesses of SnO_2_ film and glass were 500 nm and 0.5 µm, respectively, and the samples size was 15 mm × 20 mm.

### Plasma treatment

The AP-ICP source used in this study was designed similarly with that was used in the previous study^27^ with a longer slit size (20 × 2 mm), and its schematic is shown in Fig. 1a. The plasma source consisted of a 200-mm-high L-shaped discharge quartz tube with a three-turn Cu coil and a long floating metal wire placed inside. The L-shaped discharge tube having a slit at the tube bottom was used to generate a large-area plasma. The plasma was produced using a VHF power supply (100 MHz, Nihon Koshuha HFS-100-002). H_2_/Ar mixture gas that H_2_ gas concentration was 0.05%, was flowed into the discharge tube to generate plasma with a flow rate of 6 slm. A processing chamber was used to avoid the re-oxidation of Sn particles from air. The pressure of chamber was remained at 0.7 atm (near atmospheric pressure). Figure 1b exhibits the photograph for side view and front view of plasma.

### Plasma diagnostics

The optical emission spectra of the floating wire-assisted plasma including the emission with the wavelength from 200 to 850 nm, H_α_ emission, H_β_ emission, and the emission of OH molecular band were measured using a spectrometer (Andor, SR-500-B10). The measured point was on SnO_2_ film surface that is 3 mm distance from the center of the discharge tube bottom. Gas temperature was assumed as rotational temperature^33,34,39^. The rotational temperature of OH was obtained by fitting the experiment spectra with the simulated spectral profiles of OH using LIFBASE program^40^. H radical density produced from H_2_/Ar plasma source was measured using vacuum ultraviolet absorption spectroscopy (VUVAS)^28–30^. The micro hollow cathode lamp (MHCL) used pulsed power (12 A) to generate an atmospheric-pressure plasma and generate the vacuum ultra violet (VUV) signal to monochromator and PMT detector. The absorption rate was determined from the difference between the incident intensity and absorbed intensity that were recorded by an oscilloscope (GW Instex, GDS-3504).

### Characterization

Microstructures and elemental composition of pristine sample (SnO_2_/glass) and plasma-treated samples were characterized by a cold field-emission scanning electron microscope and an energy dispersive spectrometer (Hitachi, SU-8230, FE-SEM/EDS). The Sn sphere areas and Sn particle sizes were calculated by an image analyzer (ImageJ program) from SEM images.

## Supplementary information

Supplementary file 1.

## Data Availability

The datasets generated during and/or analyzed during the current study are available from the corresponding author on reasonable request.
