# Supplementary material for: Formation of spherical Sn particles by reducing SnO2 film in floating wire-assisted H2/Ar plasma at atmospheric pressure
Source: Sci Rep. 2020 Oct 20;10:17770. doi: 10.1038/s41598-020-74663-z (PMC7576790; doi:10.1038/s41598-020-74663-z)
Supplement: Supplementary file 1 [file 41598_2020_74663_MOESM1_ESM.docx]

Supplementary Information

**Formation of spherical Sn particles by reducing SnO_2_ film in floating wire-assisted H_2_/Ar plasma at atmospheric pressure**

Thi-Thuy-Nga Nguyen^1,*^, Minoru Sasaki^2^, Takayoshi Tsutsumi^1^, Kenji Ishikawa^1^, and Masaru Hori^1^

^1^Nagoya University, Nagoya, 464-8601, Japan

^2^Toyota Technological Institute, Nagoya, 468-8511, Japan

[^*^nguyen@plasma.engg.nagoya-u.ac.jp](mailto:*nguyen@plasma.engg.nagoya-u.ac.jp)

Keywords: floating wire-assisted atmospheric-pressure plasma, atmospheric-pressure inductively couple plasma, spherical Sn particles, SnO_2_ reduction


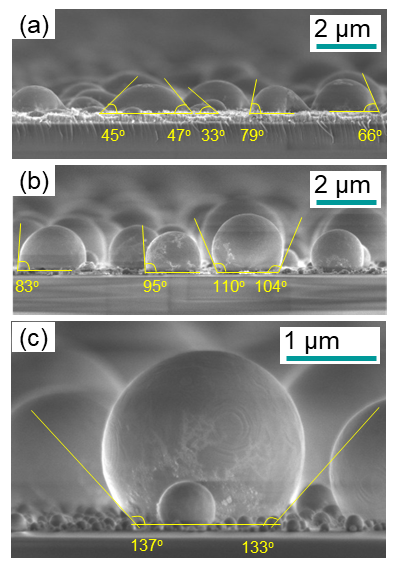


Figure S1: Cross-sectional SEM images of various Sn contact angles of treated SnO_2_/glass substrate at (a) Step 1, (b) Step 2, and (c) Step 3.


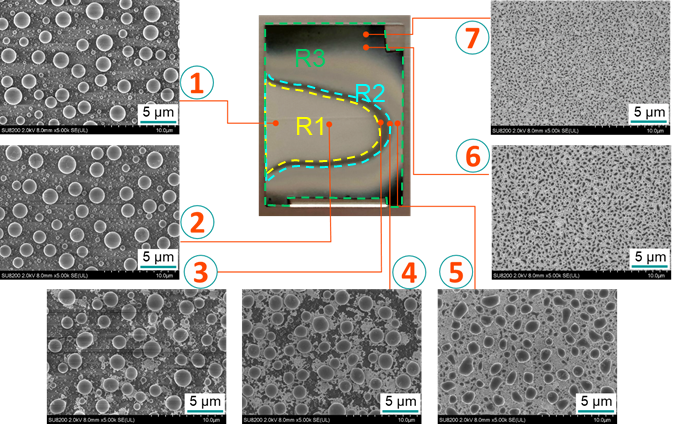


.

Figure S2: Top-view SEM images of morphology distribution of SnO_2_/glass substrate after 5 min plasma treatment without using heater.

Figure S3: Electron density of remotely floating wire-assisted H_2_/Ar plasma as a function of VHF power.

T_g_ = 940 K

Figure S4: Emission spectra including experiment data and simulation spectra of OH molecular band produced from H_2_/Ar plasma at 150 W.


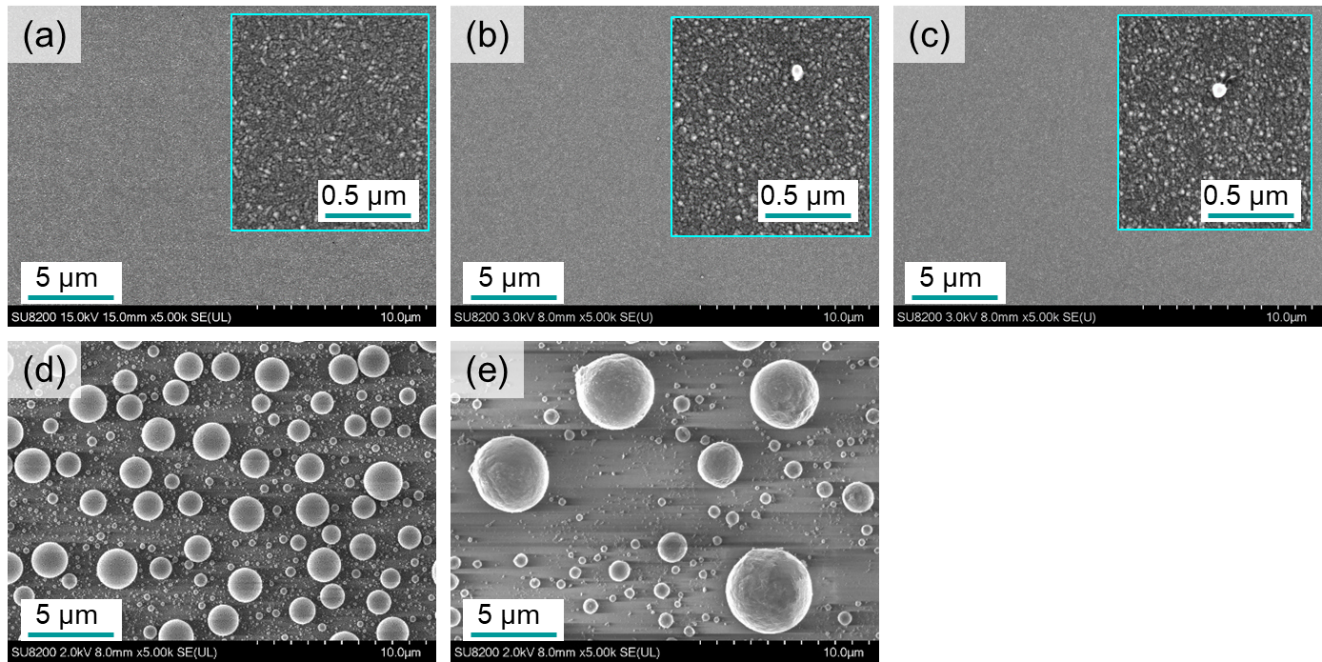


Figure S5: Comparison between samples w/o and with plasma treatment (a) pristine sample, (b) sample treated in H_2_/Ar gas at 460 ^o^C for 2 min, (c) sample treated in H_2_/Ar gas at 460 ^o^C for 20 min, (d) sample treated in H_2_/Ar plasma without using heater for 2 min, and (e) sample treated in H_2_/Ar plasma at 490 ^o^C for 2 min.
